# Activin-A Induces Fewer, but Larger Osteoclasts From Monocytes in Both Healthy Controls and Fibrodysplasia Ossificans Progressiva Patients

**DOI:** 10.3389/fendo.2020.00501

**Published:** 2020-07-14

**Authors:** Ton Schoenmaker, Esmée Botman, Merve Sariyildiz, Dimitra Micha, Coen Netelenbos, Nathalie Bravenboer, Angele Kelder, E. Marelise W. Eekhoff, Teun J. De Vries

**Affiliations:** ^1^Department of Periodontology, Academic Centre for Dentistry Amsterdam (ACTA), University of Amsterdam and Vrije Universiteit, Amsterdam, Netherlands; ^2^Department of Internal Medicine Section Endocrinology, Amsterdam Movement Sciences, Amsterdam UMC, Vrije Universiteit Amsterdam, Amsterdam, Netherlands; ^3^Department of Clinical Genetics, Amsterdam Movement Sciences, Amsterdam UMC, Vrije Universiteit Amsterdam, Amsterdam, Netherlands; ^4^Department of Clinical Chemistry, Amsterdam UMC, Vrije Universiteit Amsterdam, Amsterdam, Netherlands; ^5^Department of Hematology, Amsterdam UMC, Vrije Universiteit Amsterdam, Amsterdam, Netherlands

**Keywords:** fibrodysplasia ossificans progressiva, Activin-A, *ACVR1*, CD14+ monocyte, osteoclast

## Abstract

Fibrodysplasia Ossificans Progressiva (FOP) is a rare genetic disease characterized by heterotopic ossification (HO) that occurs in muscle tissue, tendons, and ligaments. The disease is caused by mutations in the Activin receptor type I (ACVR1) gene resulting in enhanced responsiveness to Activin-A. Binding of this molecule to the mutated receptor induces HO. Bone metabolism normally requires the coupled action of osteoblasts and osteoclasts, which seems to be disturbed during HO. We hypothesize that Activin-A may also counteract the formation of osteoclasts in FOP patients. In this study we investigated the effect of Activin-A on osteoclast differentiation of CD14+ monocytes from FOP patients and healthy controls. The lymphocytic and monocytic cell populations were determined by FACS analysis. Expression of the mutated R206H receptor was assessed and confirmed by allele specific PCR. The effect of Activin-A on osteoclastogenesis was assessed by counting the number and size of multinucleated cells. Osteoclast activity was determined by culturing the cells on Osteo Assay plates. The influence of Activin-A on expression of various osteoclast related genes was studied with QPCR. Blood from FOP patients contained similar percentages of classical, intermediate, or non-classical monocytes as healthy controls. Addition of Activin-A to the osteoclastogenesis cultures resulted in fewer osteoclasts in both control and FOP cultures. The osteoclasts formed in the presence of Activin-A were, however, much larger and more active compared to the cultures without Activin-A. This effect was tempered when the Activin-A inhibitor follistatin was added to the Activin-A containing cultures. Expression of osteoclast specific genes Cathepsin K and TRAcP was upregulated, gene expression of osteoclastogenesis related genes M-CSF and DC-STAMP was downregulated by Activin-A. Since Activin-A is a promising target for inhibiting the formation of HO in FOP, it is important to know its effects on both osteoblasts and osteoclasts. Our study shows that Activin-A induces fewer, but larger and more active osteoclasts independent of the presence of the mutated ACVR1 receptor. When considering FOP as an Activin-A driven disease that acts locally, our findings suggest that Activin-A could cause a more pronounced local resorption by larger osteoclasts. Thus, when targeting Activin-A in patients with neutralizing antibodies, HO formation could potentially be inhibited, and osteoclastic activity could be slightly reduced, but then performed dispersedly by more and smaller osteoclasts.

## Introduction

Fibrodysplasia ossificans progressiva (FOP) is a autosomal dominant severe genetic disease characterized by heterotopic bone formation where muscles, tendons, and ligaments are being converted into bone (1–3). Heterotopic ossification (HO) in FOP might appear after a flare-up, during inflammation, following injury or spontaneously. The new extra-skeletal bone is formed by endochondral ossification (1), the metabolism and composition of this heterotopic bone formed in FOP patients appears to be comparable to skeletal bone in healthy subjects. It ultimately connects to the existing skeleton, hereby gradually causing irreversible movement impairments throughout the body (4).

FOP is caused by mutations in the gene encoding the Activin receptor type 1/activin kinase 2 (ACVR1/ALK2), a bone morphogenetic protein (BMP) type 1 receptor (2, 5). Upon binding of different ligands of the TGF-β superfamily, ACVR1 receptor dimers normally form a complex with a BMP type 2-receptor dimer stimulating the SMAD1/5/8 pathway and thereby osteogenesis. The most frequent mutation causing FOP is the single nucleotide c.617G>A mutation resulting in the replacement of the amino acid arginine by histidine (R206H). This replacement stimulates osteogenic differentiation of osteoblast-like cells by causing both decreased binding of the ACVR1-inhibitor FK binding protein 12 (FKBP12) (6, 7) and increased responsiveness to BMP4 (8–10). Recently Activin-A, a TGF- β superfamily ligand that normally inhibits BMP signaling through ACVR1 (11) has been shown to induce osteogenic differentiation via the mutated receptor. In FOP-patient derived induced pluripotent stem cells (12) as well as in a mouse model of FOP (13) Activin-A was specifically shown to signal through the canonical BMP-pSMAD1/5/8 pathway, thus stimulating osteogenesis. FOP mice that received an inhibitory antibody against Activin A were protected for HO formation, holding promise for therapies addressing Activin A activity (13, 14). Recently the first results from the LUMINA-1 trial using the Activin-A antibody Garetosmab indeed showed a reduction in the formation of new lesions in patients. This study also showed a small decrease in bone lesion volume, suggesting that osteoclasts could be activated in this process (https://newsroom.regeneron.com/index.php/news-releases/news-release-details/regeneron-announces-encouraging-garetosmab-phase-2-results).

Over the past years, research on FOP has focused on the osteogenic properties of osteoblast-like cells harboring the R206H mutation. Endochondral ossification as well as normal bone remodeling however, requires the coupled action of both the bone forming osteoblasts as well as the bone resorbing osteoclasts (15, 16). Therefore, it is pivotal to investigate the potential role of the ACVR1 R206H mutation on osteoclast formation as well as the influence of Activin-A on this process.

Osteoclasts are multinucleated cells that arise through fusion of CD14 positive (CD14+) monocytic cells (17). This fusion is mediated by Macrophage Colony Stimulating Factor (M-CSF) and Receptor Activator of Nuclear factor Kappa-B Ligand (RANK-L). These molecules are normally produced by, amongst others, osteoblast like cells when bone resorption is required (15). Our group previously described the use of human periodontal ligament fibroblasts (PLF) from healthy controls and FOP patients as osteoblast-like cells. When these PLF were cocultured with non-mutated CD14+ osteoclast precursors, no significant difference in PLF induced osteoclastogenesis between the control and FOP PLF was observed (18). In addition, adding Activin-A to these cocultures inhibited osteoclast formation regardless of the mutation in the PLF cells (19). This suggests a direct effect of Activin-A on the CD14+ osteoclast precursors. All these experiments however, were performed with CD14+ not bearing the mutation of ACVR1. Monocytes from FOP patients might respond differently to Activin-A.

Elucidating the effect of Activin-A on human osteoclastogenesis in cells bearing the ACVR1-R206H mutation is especially relevant since the first clinical trial in FOP using Activin-A blocking antibodies is currently in phase II.

In this study we investigated osteoclast formation from CD14+ cells from healthy controls and FOP patients, and the potential effect of Activin-A on this osteoclastogenesis. Since the CD14+ cells express ACVR-1 (19), we hypothesize that Activin-A interacts more strongly with FOP-patient derived CD14+ cells resulting in a stronger inhibition of osteoclast formation in FOP patients compared with the healthy controls.

## Materials and Methods

### Flow Cytometry

Blood was drawn from six sex and age matched controls and patients (2 males, 4 females, age range 20–68 years, maximal age difference between control and FOP 2 years). Five of the FOP patients harbor the R206H mutation, one patient harbors a variant mutation (Q207E). This Q207E mutation is adjacent to the classical R206H mutation and also located in the GS domain. Patients with this mutation show comparable phenotypes with the R206H patients (20, 21). Written informed consent was obtained from all donors as required by the Medical Ethics Review Committee of the Amsterdam UMC, Vrije Universiteit Amsterdam (research protocol 2012.467). The blood cell composition was analyzed by four color Flow Cytometry using the FACSCanto Flow Cytometry system (BD Biosciences, Franklin Lakes, NJ, USA). First the erythrocytes were lysed using Pharmlyse (BD Biosciences, Franklin Lakes, NJ, USA) and washed once with phosphate-buffered saline (PBS) containing 0.1% human serum albumin. The cells were incubated for 15 min at room temperature with either fluorescein isothiocyanate (FITC), phycoerythrin (PE), peridinyl chlorophyllin (PerCP), Phycoerythrin-Cyanine 7 (PC7), allophycocyanin (APC) allophycocyanin-cyanine (APC-H7), or Krome Orange (KO) conjugated monoclonal antibodies and washed once with PBS containing 0.1% human serum albumin. The pan-leucocyte marker CD45 (KO-labeled) was used to discriminate between white blood cells and unlysed red blood cells or debris. Lymphoid markers (CD4 APC-H7, CD8 FITC, CD19 APC) were used to discriminate between T-cells (CD4, CD8), B-cells (CD19). CD8 (FITC-labeled), and CD4 (APC-H7-labeled) were included to identify the cytotoxic/suppressor T-cells and T-helper/inducer cells, respectively. CD14 (PercP-labeled) and CD16 (PE labeled) were used to discriminate between classical monocytes (CD14++CD16−), intermediate monocytes (CD14++CD16+) and non-classical monocytes (CD14+CD16+), all of which have the capacity to differentiate into osteoclasts (22). The different monoclonal antibody clones used are listed in Supplementary Table 1. Data acquisition was performed on the FACSCanto system and analysis was performed using Infincyt software (Cytognos).

### CD14+ Cell Isolation

For the initial TRAcP staining and QPCR experiments CD14^+^ monocytes were isolated from the blood from 6 FOP patients and sex- and age- matched controls (20–40 ml blood per donor), for all other experiments human buffy coats (Sanquin, The Netherlands) or blood from healthy donors was used. The CD14+ cells were isolated as described before (23).

Briefly, peripheral blood mononuclear cells were isolated using Ficoll-Paque density gradient centrifugation. Subsequently cells were incubated with CD14-antibody tagged microbeads (Miltenyi Biotec, Bergisch Gladbach, Germany) and sorted using a manual MACS magnetic cell sorter (Miltenyi Biotec) according to the manufacturer's instructions (24). The purity of the cells was determined with flow cytometry (FACSverse™, BD Biosciences, Piscataway, USA). For the analysis, cells were incubated with FITC labeled anti-human CD14 (Miltenyi Biotec) or its equivalent isotype control IgG2a (Miltenyi Biotec) for 30 min in the dark on ice. After incubation, cells were washed to remove unbound antibodies, recovered in FACS buffer and analyzed (30 s or 100,000 viable events) on a BD Bioscience FACSverse flow cytometer. Purity was confirmed to be at least 80%.

### Osteoclastogenesis

Purified CD14+ cells were suspended in culture medium consisting of αMEM (Thermo Fisher Scientific, Waltham, MA, USA) supplemented with 10% FCS (HyClone, Logan, UT), and 1% antibiotics: 100 U/ml penicillin, 100 μg/ml streptomycin, and 250 ng/ml amphotericin B (Sigma, St. Louis, MO, USA). Cells were cultured in a 96 well-plate at a density of 1 × 10^5^ cells/well, for the first 3 days with 25 ng/ml macrophage colony-stimulating factor (M-CSF) (R&D systems, Oxon, UK), without or with 50 ng/ml Activin-A (Sigma). After 3 days the medium composition was changed to 10 ng/ml M-CSF and 2 ng/ml receptor activator of nuclear factor kappa-B ligand (RANKL) (R&D Systems), without or with 50 ng/ml Activin-A. In the blocking experiments cells were also cultured with 50 ng/ml Activin-A and 500 ng/ml follistatin (R&D systems) according to advice from Wang (25). All cultures were maintained at 37°C in a humidified atmosphere under 5% CO_2_ for 21 days_._ Culture media were replaced every 3–4 days.

In the experiments with CD14+ cells from control and FOP, blood from 6 controls and 6 FOP patients were used. For the TRAcP staining all experimental conditions were plated in triplicate. For the counting of the osteoclasts five designated fields per well were counted and the number of TRAcP positive multinuclear cells (>3 nuclei) were counted. For the follistatin experiments blood from 3 healthy donors was used and each experimental condition was plated in quadruplicate. Counting of osteoclasts was performed as described above. For the size of the osteoclasts four low magnification (10X) pictures per well were taken at designated fields with a digital camera (Leica, Wetzlar, Germany). Subsequently the size was measured using Image-Pro Plus (MediaCybernetics, Rockville, USA). Osteoclast size is depicted in um^2^ per osteoclast or as the percentage of surface area that is occupied by the osteoclasts.

### TRACP Staining

Tartrate Resistant Acid Phosphatase (TRACP) staining was performed with the Leukocyte Acid Phosphatase Staining Kit (Sigma, St. Louis, MO, USA) as previously described (18, 19). Nuclei were stained with diamidino-2 phenylindole dihydrochloride (DAPI). For the counting of the osteoclasts five designated fields per well were selected and the number of TRAcP positive multinuclear cells (>3 nuclei) were counted.

### RNA Isolation and Real-Time Quantitative Polymerase Chain Reaction (Q-PCR)

Cells were cultured for 7, 14, and 21 days in 96 well-plates. For each experimental condition 3 wells were pooled.

RNA was isolated using the RNeasy mini kit (QIAGEN, Hilden, Germany) following manufacturer's instructions. The reverse transcriptase reaction was performed with the first strand cDNA synthesis kit (Thermo Fisher Scientific) according to the manufacturers protocol, using both the Oligo(dT)18 and the D(N)6 primers.

Allele specific PCR primers were used to distinguish between c.617G>A and non-mutated ACVR-1. Primers were described by Kaplan et al. (26). To detect the FOP allele 300 nM of the *ACVR1 c.617A* primers were used in a standard two step QPCR program with an annealing temperature of 63°C. To detect the control allele 150 nM of the *ACVR1 c.617G* primers were used in a standard two step QPCR program with an annealing temperature of 63°C. For the other genes Q-PCR primers were designed using Primer Express software, version 2.0 (Applied Biosystems, Foster City, CA, USA) (Table 1). To avoid amplification of genomic DNA, each amplicon spanned at least one intron. Q-PCR was performed on the LC480 light cycler (Roche, Basel, Switzerland). Three nanogram cDNA was used in a total volume of 20 μl containing Light Cycler SybrGreen1 Master mix (Roche) and 1 μM of each primer. A standard two step QPCR program with an annealing temperature of 60°C was performed. Sequence information for all primers are listed in Table 1. Expression of housekeeping gene porphobilinogen deaminase (*PBGD*) was not affected by the experimental conditions. Samples were normalized for the expression of *PBGD* by calculating the ΔCt (Ct_, *geneofinterest*_–Ct_, *PBGD*_) and expression of the different genes was expressed as 2^−(ΔCt)^. All qPCRs had equal efficiencies.

**Table 1:** Primer sequences used for quantitative PCR.

**Gene**	**Sequence 5^**′**^-3**	**Amplicon** **length (bp)**	**Ensemble gene ID**
*PBGD*	TgCAgTTTgAAATCATTgCTATgTC	84	ENSG00000113721
	AACAggCTTTTCTCTCCAATCTTAgA		
*ACVR1 c.617G*	TggTACAAAgAACAgTggCTAg	101	ENSG00000115170
	CCATACCTgCCTTTCCCgA		
*ACVR1 c.617A*	TggTACAAAgAACAgTggCTTA	101	
	CCATACCTgCCTTTCCCgA		
*ACVR1*	CAgCTgCCCACTAAAggAAAAT	68	
	AATAATgAggCCAACCTCCAAgT		
*CSF1*	CCgAggAggTgTCggAgTAC	100	ENSG00000184371
	AATTTggCACgAggTCTCCAT		
	CTCggAgCTCTgATgTgTTgAA		
*DCSTAMP*	ATTTTCTCAgTgAgCAAgCAgTTTC	101	ENSG0000016493
	AgAATCATggATAATATCTTgAgTTCCTT		
*ID-1*	ACgTgCTgCTCTACgACATgA	56	ENSG00000125968
	TgggCACCAgCTCCTTgA		
*TRAcP*	CACAATCTgCAgTACCTgCAAgAT	128	ENSG00000102575
	CCCATAgTggAAgCgCAgATA		
*CTSK*	CCATATgTgggACAggAAgAgAgTT	149	ENSG00000143387
	TgCATCAATggCCACAgAgA		
*NFATc1*	AgCAgAgCACggACAgCTATC	143	ENSG00000131196
	ggTCAgTTTTCgCTTCCATCTC		
*AlphaV Integrin*	TACAgCAggTCCCCAAgTCACT	100	ENSG00000138448
	AATTCAgATTCATCCCgCAgAT		

*PBGD, porphobilinogen deaminase; ACVR1c.617G, Activin A receptor type I, control allele; ACVR1c.617A, Activin A receptor type I, FOP allele; ACVR1, Activin A receptor type I; CFS1, colony-stimulating factor1 [coding for macrophage-colony stimulating factor (M-CSF)]; DC-STAMP, dendritic cell-specific transmembrane protein; ID-1, Inhibitor of DNA binding 1; protein; TRAcP, tartrate resistant acid phosphatese; CTSK, Cathepsin-K; NFATc1, nuclear factor of activated T-cells 1; Alpha V Integrin, Integrin subunit Alpha V. For each gene, the first oligonucleotide sequence represents the forward primer, the second sequence the reverse primer*.

### Osteoclast Activity on Osteo Assay Surface

CD14+ cells were isolated from blood from 4 healthy donors as described in the CD14+ cell isolation section. Osteoclastogenesis was performed on 96 well-Osteo Assay surface plates (Corning Costar, Lowell, MA, USA) as described in the osteoclastogenesis section either without or with Actvin-A and with both Activin-A and Follistatin. Each experimental condition was plated in quadruplicate. Cells were cultured for 14 and 21 days. To visualize the lysis of the calcium phosphate coating, wells were incubated for 5 min in 10% bleach, washed with H_2_O and air dried. For the quantification of the total resorbed area, 4 pictures were taken per well at 10 x magnification. Resorbed area was measured using Image-Pro Plus (MediaCybernetics, Rockville, USA) and depicted as percentage of resorption per well.

### Statistical Analysis

Differences between cultures without and with Activin-A were tested using the Wilcoxon matched-pairs signed rank test. For differences between Control and FOP cultures the Mann-Whitney test was used. In the follistatin experiments the Friedmans test with the Dunn's multiple comparison test was used. Differences were considered to be significant when the *p*-value was lower than 0.05.

## Results

### No Difference in Lymphocyte and Monocyte Subsets Between Controls and FOP Patients

**Figure 1:**
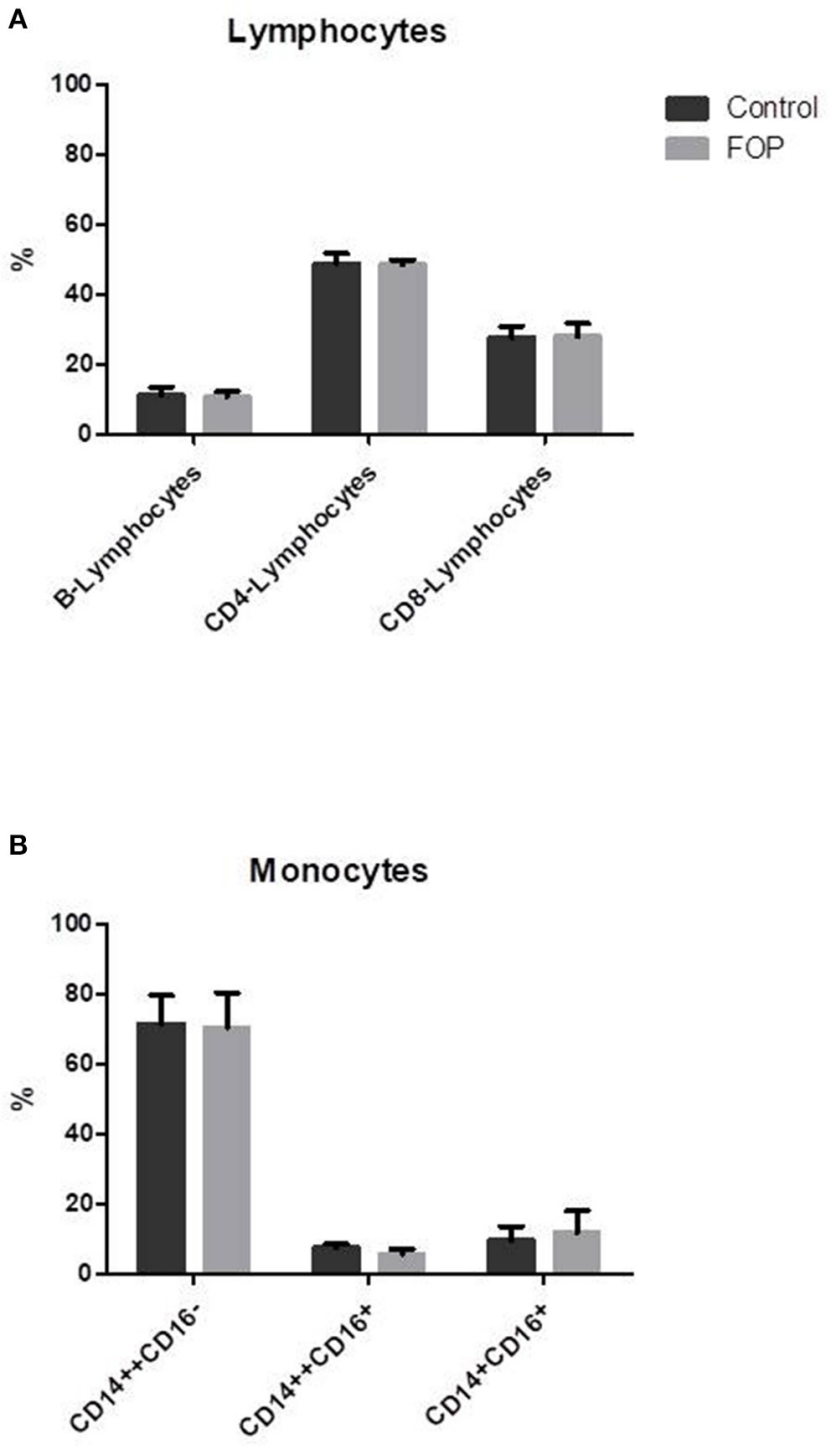
Blood cell composition does not differ between control and FOP blood. Control or FOP blood cell populations were analyzed with FACS analysis. There are no significant differences in the percentages of B-lymphocytes, CD4-lymphocytes, or CD8-lymphocytes between control and FOP blood **(A)**. The percentage of classical CD14++CD16−, intermediate CD14++CD16+ and non-classical CD14+CD16+ was also similar between control and FOP blood **(B)**. *n* = 6 for both control and FOP (unpaired *t*-test).

Although CD14+ cells were isolated for subsequent osteoclastogenesis experiments, blood composition was first determined, since cellular composition of peripheral blood may prime osteoclast precursors [Reviewed in (27)]. No difference was found between the percentage B-lymphocytes and CD4 or CD8 positive T-lymphocytes between the control and FOP blood samples (Figure 1A). Also, there were no differences in the total CD14 positive monocytic cell populations. Since osteoclasts differentiate from CD14 positive cell populations, and different CD14 cell populations show distinct osteoclastogenesis dynamics (23), we next investigated the CD14 cell composition in the blood of the controls and FOP patients. There were no differences between classical monocytes (CD14++CD16−), intermediate monocytes (CD14++CD16+) and non-classical monocytes (CD14+CD16+) between controls and FOP (Figure 1B, Gating strategy is shown in Supplementary Figure 1).

### ACVR-1 Is Expressed in CD14+ Monocytes; The c.617G>A FOP Allele Is Only Expressed in Monocytes From FOP Patients

To show that the CD14+ cells undergoing osteoclast differentiation indeed express the *ACVR1* gene and that only FOP monocytes from the R206H patients express the c.617G>A FOP allele we performed allele specific QPCR as described by Kaplan et al. (26), where the 3′ last nucleotide of the forward primer is complementary to either the control (c.617G) or the FOP (c.617A) allele. The second last 3′ nucleotide is a deliberate mismatch. The reverse primer is the same for both PCR reactions.

**Figure 2:**
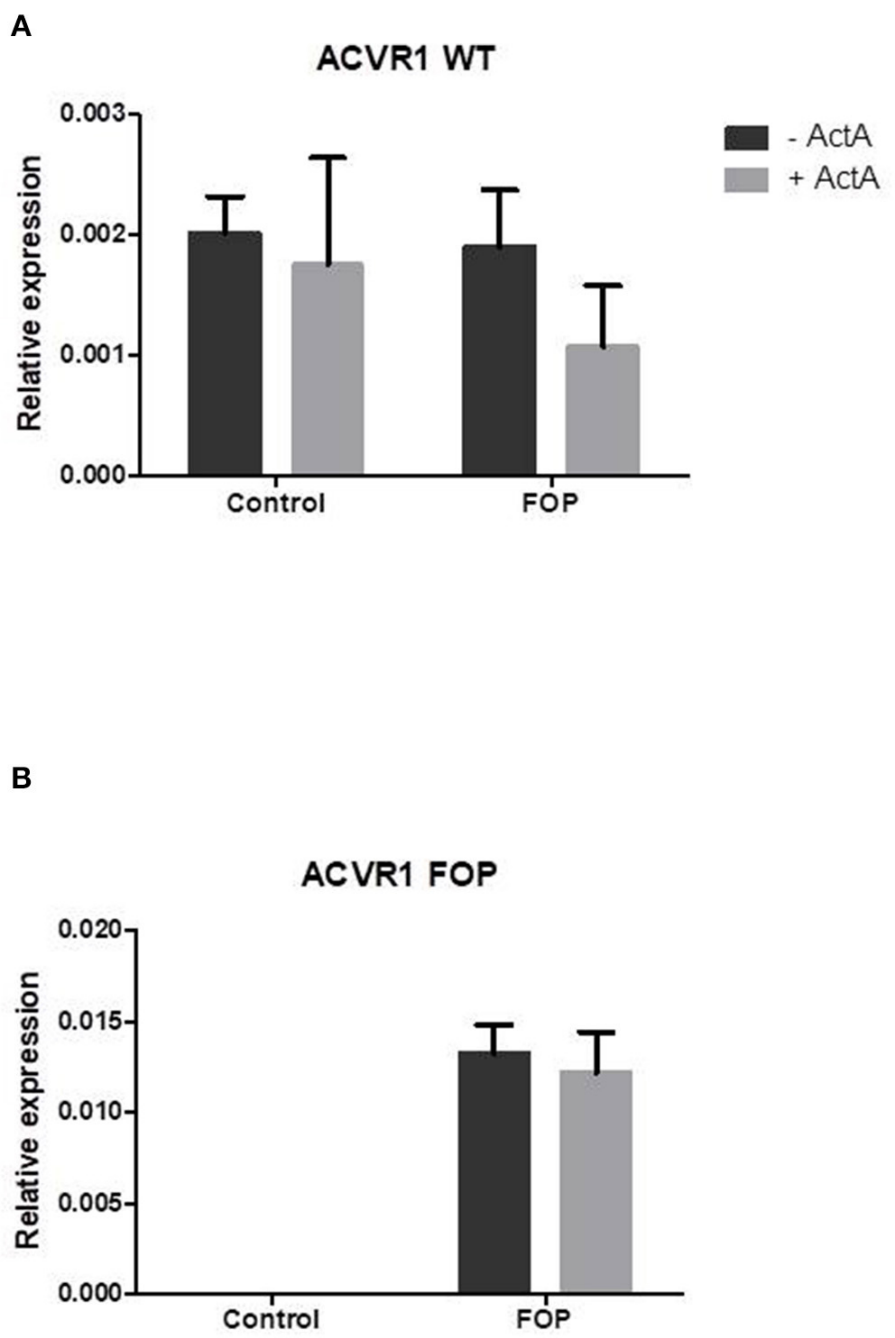
Expression of the FOP c.617G>A allele is only present in the FOP osteoclast precursors. Control or FOP CD14+ cells were cultured with M-CSF and RANK-L, without and with Activin-A 50 ng/ml. RNA was isolated after 7 days and QPCR was performed. **(A)** The control c.617G allele is equally expressed in both control and FOP cells. **(B)** The FOP c.617A allele is only expressed in FOP cells harboring the R206H mutation (the cells from the patient with the Q207E mutation did not show any expression of the c.617A allele, data not shown). Activin-A does not alter the expression of either allele. Expression was normalized based on expression of the housekeeping gene PBGD. *n* = 6 for both control and FOP.

After 7 days of culture the wild type c.617G allele is expressed in both the control and the FOP cells at similar levels (Figure 2A). The FOP c.617A allele however, is only expressed by the FOP cells (Figure 2B). Expression of ACVR1 increases from day 7–14 and stays the same to day 21 (data not shown). The expression of both the control and the FOP allele was not influenced by Activin-A (Figures 2A,B).

### Activin-A Inhibits Osteoclast Formation but Increases Osteoclast Size in Both Control and FOP Cultures

**Figure 3:**
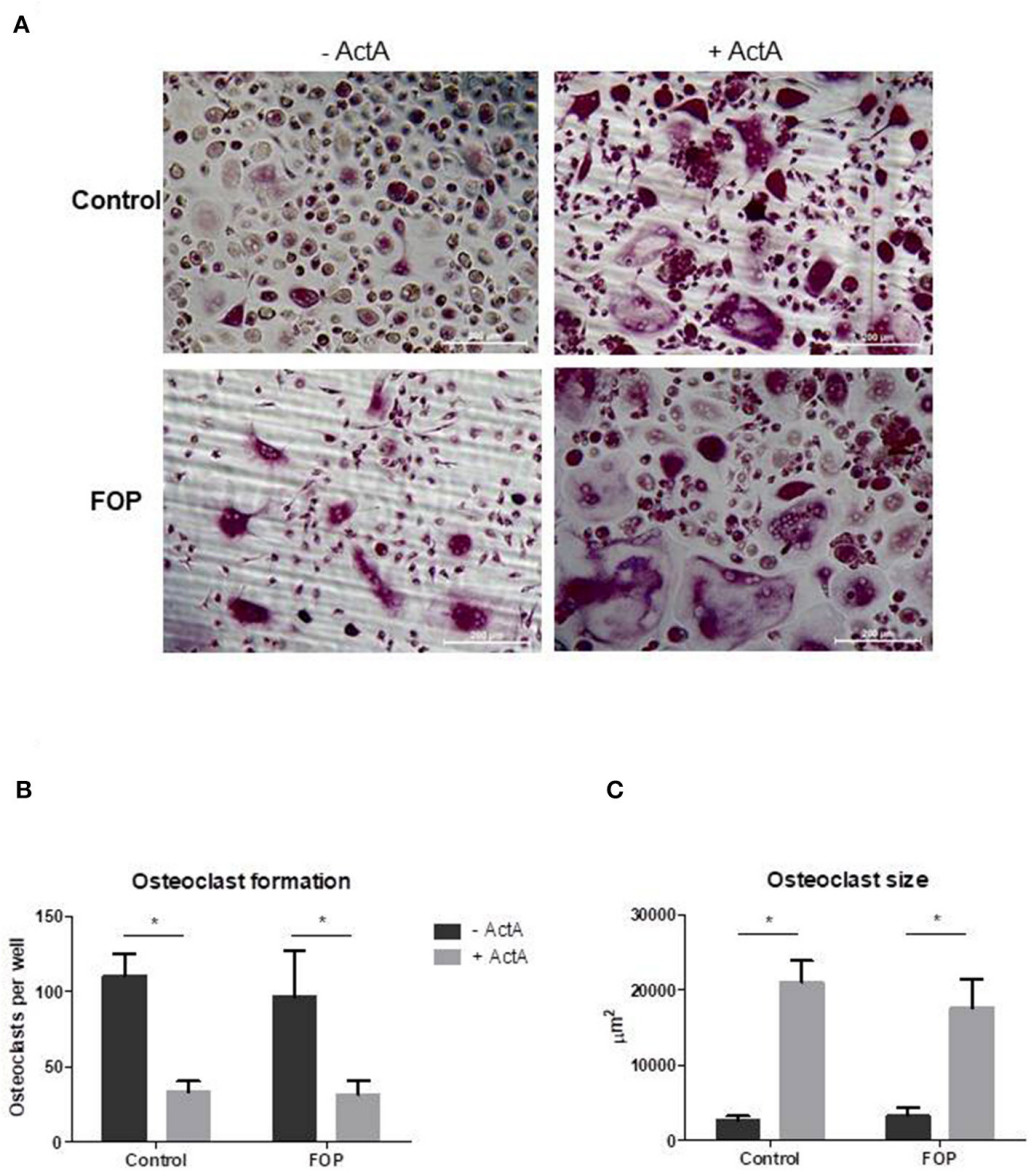
Activin-A inhibits osteoclast formation in both control and FOP cultures. Control or FOP CD14+ cells were cultured with M-CSF and RANK-L, without and with Activin-A 50 ng/ml. Cells were stained for TRAcP and nuclei were stained with DAPI after 21 days of culture. **(A)** Micrograph of the stained cultures of the four different conditions after 21 days. **(B)** Equal numbers of osteoclasts were formed in the control and FOP cultures. Activin-A significantly inhibited osteoclast formation in both cultures. **(C)** The average size of the osteoclasts was significantly increased when Activin-A was added to the cultures. *n* = 6 for both control and FOP (Wilcoxon matched-pairs signed rank test, *p ≤ 0.05).

Having shown that both control and FOP-derived osteoclast precursors express ACVR1, and that only FOP-derived precursors express the c.617G>A variant, we next investigated whether this mutation alters the effect of Activin-A on osteoclast formation. Cells were cultured with M-CSF and RANK-L without and with Activin-A. After 21 days the cells were fixed and stained for TRAcP and the nuclei were stained with DAPI. Similar numbers of osteoclasts formed in control and FOP cultures (Figures 3A,B). Although the osteoclasts were significantly larger in the presence of Activin-A, the total number of osteoclasts was significantly inhibited by this molecule in both control and FOP cultures (Figures 3A–C).

### Activin-A Alters Osteoclast Related Gene Expression in Both Control and FOP Cultures

**Figure 4:**
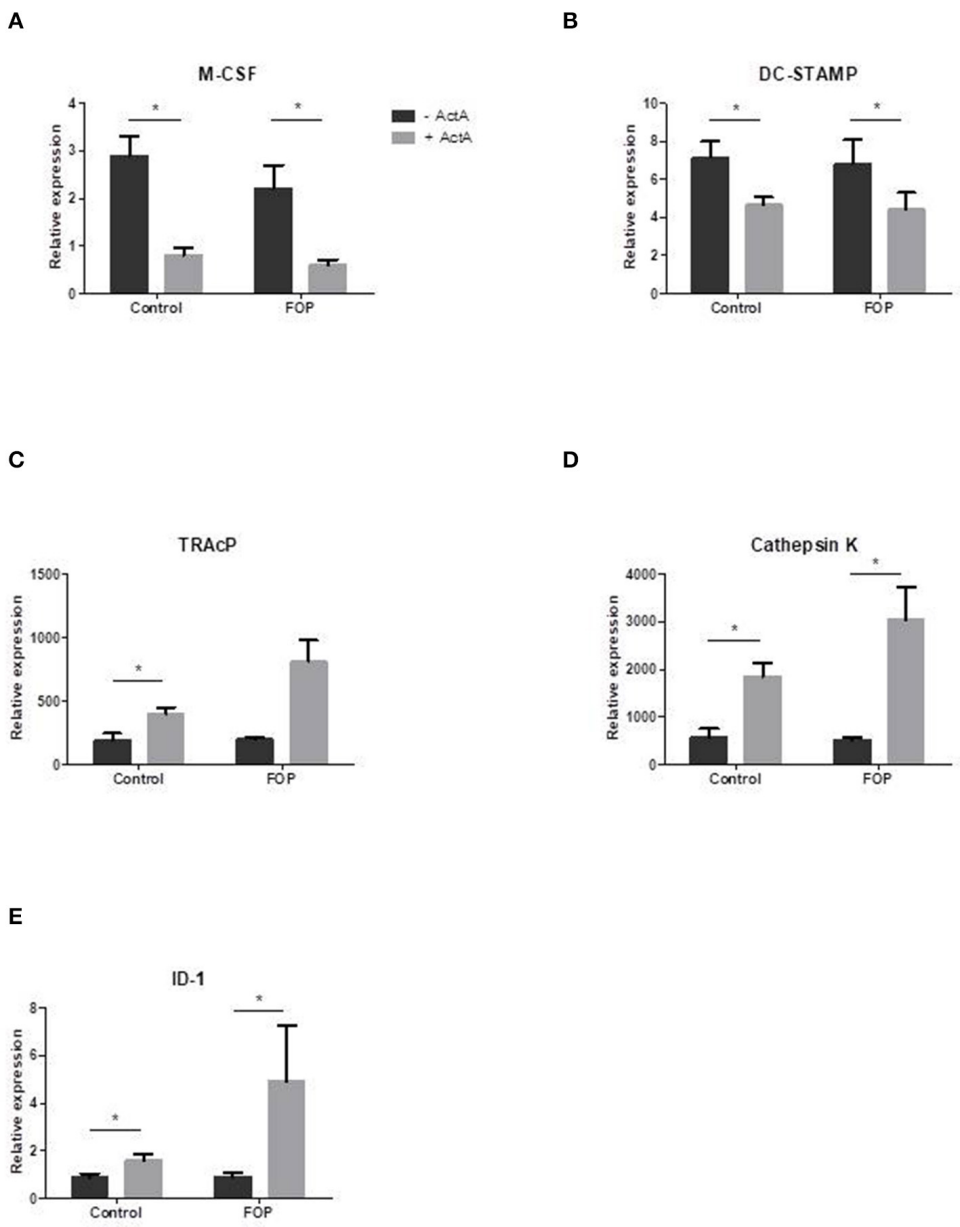
Osteoclast related gene expression is altered in the presence of Activin-A. Control or FOP CD14+ cells were cultured with M-CSF and RANK-L, without and with Activin-A 50 ng/ml. RNA was isolated after 7 and 21 days and QPCR was performed. Gene expression of tested genes was similar in control and FOP cultures. **(A)** Expression of M-CSF was downregulated after 7 days of culturing in the presence of Activin-A. **(B)** Expression of DC-STAMP was downregulated after 7 days of culturing in the presence of Activin-A. **(C)** Expression of TRAcP was upregulated after 21 days of culturing in the presence of Activin-A. **(D)** Expression of Cathepsin K was upregulated after 21 days of culturing in the presence of Activin-A. **(E)** Expression of ID-1 was upregulated after 7 days of culturing in the presence of Activin-A. *n* = 6 for both control and FOP (Wilcoxon matched-pairs signed rank test, *p ≤ 0.05).

In order to elucidate the molecular mechanisms by which Activin-A inhibits osteoclast formation in these cultures we analyzed gene expression with qPCR at several time points on osteoclast related genes. Two of the key role players in osteoclast formation, M-CSF, and DC-STAMP, were significantly downregulated in the presence of Activin-A after 7 days of culture (Figures 4A,B). In contrast, ID-1, one of the target molecules of SMAD1/5/8 signaling, was significantly upregulated in both cultures after 7 days in the presence of Activin-A (Figure 4E). Osteoclast-specific genes TRAcP and Cathepsin-K were both upregulated in the presence of Activin-A after 21 days (Figures 4C,D).

### Inhibition With Follistatin Reverses the Activin-A Effect

To further prove that Activin-A results in the formation of less but larger osteoclasts, we performed inhibition experiments with follistatin, a natural inhibitor of Activin-A that can reverse Activin-A effects (25). Initial titration experiments showed that Activin-A's effect on osteoclast formation was reduced at a concentration of 500 ng/ml follistatin (data not shown). Since nor osteoclast formation, nor osteoclast-related gene expression nor the effect of Activin-A is different between control and FOP osteoclast cultures, these experiments were performed on CD14+ cells isolated from buffy coats from healthy donors.

**Figure 5:**
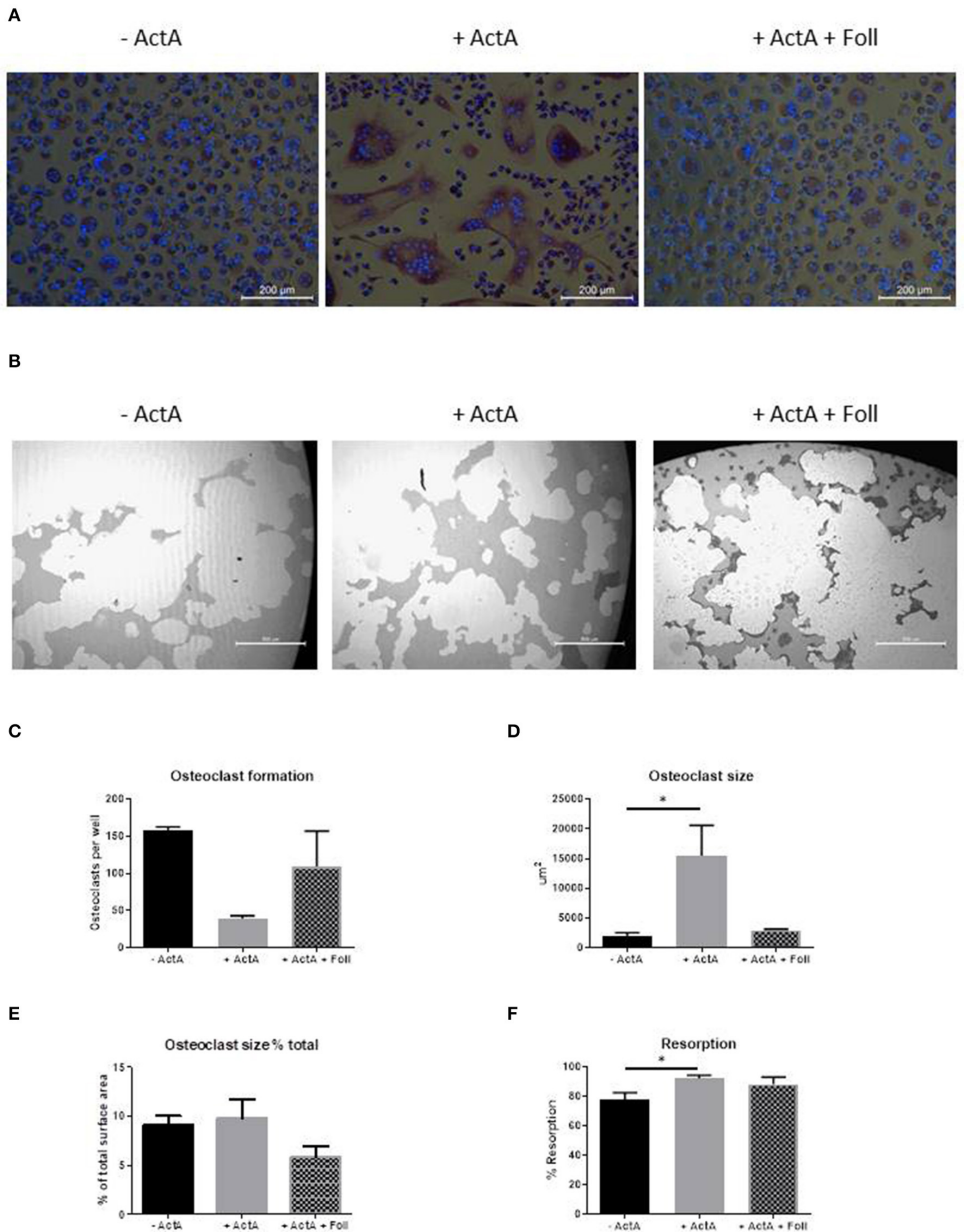
Follistatin reduces the Activin-A effect. CD14+ cells from three healthy donors were cultured with M-CSF and RANK-L, without and with Activin-A (50 ng/ml). Experiments were plated in quadruplicate. To block the Activin-A effect a third experimental condition was added where follistatin (500 ng/ml) was present in the cultures. After 21 days cells were stained for TRAcP and nuclei were stained with DAPI. **(A)** Micrographs of the stained cultures of the three different conditions. **(B)** Micrographs of the lysed calcium phosphate surface from the osteo assay plates of the three different culture conditions. **(C)** Follistatin reduces the inhibitory effect of Activin-A on the number of formed osteoclasts. **(D)** Follistatin reduces the increasing effect of Activin-A on the size of the formed osteoclasts. **(E)** The percentage of the total area occupied by the formed osteoclasts does not differ between the three culture conditions. **(F)** The percentage of resorbed area in the osteo assay plates is higher when Activin-A is present in the cultures, implicating a higher activity per osteoclast. This effect is nullified when Follistatin is added. *n* = 3 (Friedmann test with Dunn's multiple comparisons, *p ≤ 0.05).

After 21 days of culturing with follistatin the inhibitory effect of Activin-A on osteoclast formation and inductive effect on size was reduced (Figures 5A,C,D). The total percentage of area covered by the osteoclasts however, did not differ between the different culture conditions (Figure 5E). Early gene expression data also show that the increased expression of NFATc1, AlphaV Integrin, TRAcP, and Cathepsin K by Activin-A is reversed by follistatin (Supplementary Figure 2).

### Activin-A Increases Overall Resorptive Activity

We next investigated the effect of Activin-A on the resorptive activity of the osteoclasts. Osteoclastogenesis was performed with CD14+ cells from buffy coats from healthy donors on Osteo Assay plates (Corning Costar) in the absence or presence of Activin-A or with both Activin-A and Follistatin. The lysis of the inorganic calcium phosphate coating was used as a measure of the resorptive activity of the osteoclasts. In line with the finding that the osteoclasts are larger in the presence of Activin-A, also the total percentage of resorbed area was increased in this culture condition (Figures 5B,F).

## Discussion

Heterotopic bone from FOP patients displays similar histological bone parameters such as osteoblast and osteoclast activity as normal skeletal bone. This assumes that remodeling of heterotopic bone is comparable to that of normal bone, but this has not been extensively investigated. One of the recent discoveries is the activating effect of Activin-A specifically on the mutated ACVR1 receptor that induces HO in muscle, tendons, and ligaments (12, 13). The effect of Activin-A on osteoclast formation from monocytes from FOP patients has not been previously investigated. In this study we show for the first time that Activin-A induces fewer but larger osteoclasts from both control and FOP derived human monocytes. Studies using murine cells have reported contradictory findings on the effect of Activin-A on osteoclasts, probably depending on the source of osteoclast precursors used. Some groups show that Activin-A enhances osteoclast formation and activity when using murine whole bone marrow cultures or the mouse macrophage cell line RAW246.7 (28–30). In contrast, Fowler et al. (31) showed that Activin-A suppressed osteoclastogenesis when using stromal cell depleted murine bone marrow macrophages.

We show that control as well as FOP monocytes expressed ACVR-1 at a similar level and only the FOP monocytes expressed the mutated variant of this gene, as expected. This was tested for the 5 out of 6 patients, bearing the classical R206H mutation. Expression of the rarer mutant Q207E, which is one amino acid further on the ACVR-1 protein, was not assessed. At the biochemical level, binding of osteogenic signaling inhibitor FKBP12 is less efficient in both R206H and Q207E. We therefore have reasons to assume that the addition of Activin-A may result in similar effects in both mutations.

Given the expression of the mutated version of ACVR1 in FOP monocytes, which seemed higher even than the unaffected allele, we hypothesized that Activin-A would have a more profound effect on the mutated CD14 cells compared to the control cells. However, we did not observe any difference in osteoclastogenesis between the control and FOP cultures. The addition of Activin-A had an inhibitory effect on the number of osteoclasts. However, these fewer osteoclasts were on average 5–8-fold larger in both cultures, suggesting this effect is not mediated via the mutated receptor. Binding of Activin-A to ACVR1 normally inhibits BMP signaling via this receptor (13, 32). In contrast to the generally accepted role of BMPs in osteogenesis, several studies have shown that BMP signaling is also important for osteoclast formation. Inhibition of this signaling, either via deletion of BMPR2 (33) or by inhibiting SMAD 4 or SMAD 1/5 (34, 35) reduces DC-STAMP expression and inhibits osteoclastogenesis. Similarly, the inhibitory effect of Activin-A on BMP signaling could be the cause of the inhibited osteoclastogenesis seen in this study. QPCR analysis however, showed a higher expression of ID-1, a downstream target molecule of SMAD1/5/8 phosphorylation (25), in the presence of Activin-A especially in the FOP cells, suggesting that enhanced SMAD1/5/8 signaling may occur in FOP patients derived monocytes. The fact that this does not seem to influence osteoclastogenesis might be related to the timing of effects. During the early stages of osteoclastogenesis signaling via the non-canonical BMP pathway seems to be more important, whereas during the later stages the canonical signaling pathway seems to play a more important role (33). The lower expression of M-CSF and DC-STAMP by Activin-A could explain the inhibition of osteoclastogenesis. Next to an inhibitory effect on osteoclast formation we also observed that the presence of Activin-A induced larger osteoclasts. Osteoclast precursors with lower expression of the fusion receptor DC-STAMP have been shown to give rise to higher TRAcP expression and bigger osteoclasts compared to precursors with higher DC-STAMP expression (36). This could be the explanation for our observed difference in osteoclast size, since we also see a decreased DC-STAMP and increased TRAcP expression in the presence of Activin-A. Omi et al. recently reported that ACVR1 plays a role in osteoclast formation via BMP7 induced canonical SMAD signaling pathways (37). They also showed that signaling via BMPR1A seems to be more important for the fusion of osteoclast precursors. Possibly the observed increase in osteoclast size in our experiments is due to signaling of Activin-A via a BMPR1A receptor complex, probably bypassing ACVR-1. The correlation between osteoclast size and activity is not entirely clear. In some cases giant osteoclasts seem to be an indication of less active osteoclasts. This is especially apparent when osteoclast activity is inhibited by bisphosphonates. In other pathological conditions such as Paget Disease, the giant osteoclasts are also highly active (38, 39). A positive correlation between size and activity has been described by Piper et al., who correlated activity to the number of nuclei per osteoclast (40). We could later confirm this in another way, by correlating osteoclast area to actin ring surface (41). We showed that osteoclast size is proportional to the number of actin rings per osteoclast and that the percentage of actin ring area per osteoclast is relatively constant, being ~20%. Together, these two studies imply that larger osteoclasts are more active in resorption, albeit that per osteoclast a constant area is used for resorption. Our results, less, but larger and more active osteoclast when cultured with Activin-A that cover a similar area as the more but smaller osteoclasts without Activin-A, also suggest that indeed the larger osteoclasts are more active, but that this activity is performed more localized since the the area covered by osteoclasts is the same in the two conditions. In the context of Activin-A, it is likely the local *in vivo* circumstances that determine overall osteoclast activity. Upadhyay et al. (42) recently described a partial resorption of HO after treatment with Activin-A antibodies in their FOP mouse model. This implies that osteoclast function is inhibited by local access of Activin-A and that the osteoclast function can be restored after Activin-A antibody treatment. Our results, with the obvious limitation of the cell biological approach using CD14+ cells and differentiation factors M-CSF and RANKL in the presence of Activin A, suggest an increase of resorption. Whether inhibition of Activin-A *in vivo* may have different effects, cannot be ruled out. In conjunction with the heterotopic bone formation potential of Activin A in FOP, our results rather show that Activin-A may contribute to an increased bone metabolism altogether.

Our study is the first to investigate the effect of Activin-A on human FOP-patient derived osteoclasts. We demonstrated that Activin-A induces fewer but larger osteoclasts irrespective of the presence of the mutated ACVR1 receptor, but further studies on FOP-patients derived cells are necessary for understanding the full width of the mode of action of Activin-A. This is even more important in the light of promising ongoing clinical trials in FOP that specifically target mutant ACVR-1 in general or Activin-A in particular (43). It remains intriguing that Activin A only causes heterotopic bone formation, and only in FOP, leaving the normal skeleton seemingly untouched. Likewise, it is conceivable that osteoclasts that remodel heterotopic bone, could respond differently to anti-Activin A in the microenvironment of heterotopic bone. We propose that when inhibiting osteogenesis by anti-Activin A in a heterotopic bone context, osteoclast activity could be reduced but more dispersed since it is performed by more but smaller osteoclasts.

## Data Availability Statement

The datasets generated for this study are available on request to the corresponding author.

## Ethics Statement

The studies involving human participants were reviewed and approved by Medical Ethics Review Committee of the Amsterdam UMC, Vrije Universiteit Amsterdam. The patients/participants provided their written informed consent to participate in this study.

## Author Contributions

TS designed and conducted the study, performed the data analysis and wrote the manuscript. EB, DM, CN, and NB contributed to the design of the study and writing the manuscript. MS performed the follistatin experiments. AK performed the FACS analysis. EE and TD supervised the study, contributed to the study design, and writing the manuscript. All authors contributed to the article and approved the submitted version.

## Conflict of Interest

The authors declare that the research was conducted in the absence of any commercial or financial relationships that could be construed as a potential conflict of interest.

## Supplementary Material

Supplementary Figure 1 Gating strategies for the FACS analysis. Gating strategy used to discriminate between between classical monocytes (CD14++CD16−), intermediate monocytes (CD14++CD16+) and non-classical monocytes (CD14+CD16+).

Supplementary Figure 2 Follistatin reduces the Activin-A effect on osteoclast related gene expression. CD14+ cells from pne healthy donor were cultured with M-CSF and RANK-L, without and with Activin-A (50 ng/ml). Experiments were plated in quadruplicate. To block the Activin-A effect a third experimental condition was added where follistatin (500 ng/ml) was present in the cultures. RNA was isolated after 7 days and QPCR was performed. The inductive effect of Activin-A on the gene expression of **(A)** the early transcription factor NFATc1, **(B)** the on osteoclasts abundantly expressed AlphaV integrin, **(C,D)** the osteoclast specific markers TRAcP and Cathepsin K is reduces by follistatin. *N* = 1, mRNA was isolated from one of confirmative experiments shown in Figure 5, *n* = 4 wells per condition (Friedmann test with Dunn's multiple comparisons).

Supplementary Table 1 Monoclonal antibody clones used in FACS analysis.
