# Supplementary figures and images for: Activin-A Induces Fewer, but Larger Osteoclasts From Monocytes in Both Healthy Controls and Fibrodysplasia Ossificans Progressiva Patients
Source: Front Endocrinol (Lausanne). 2020 Jul 14;11:501. doi: 10.3389/fendo.2020.00501 (PMC7371852; doi:10.3389/fendo.2020.00501)

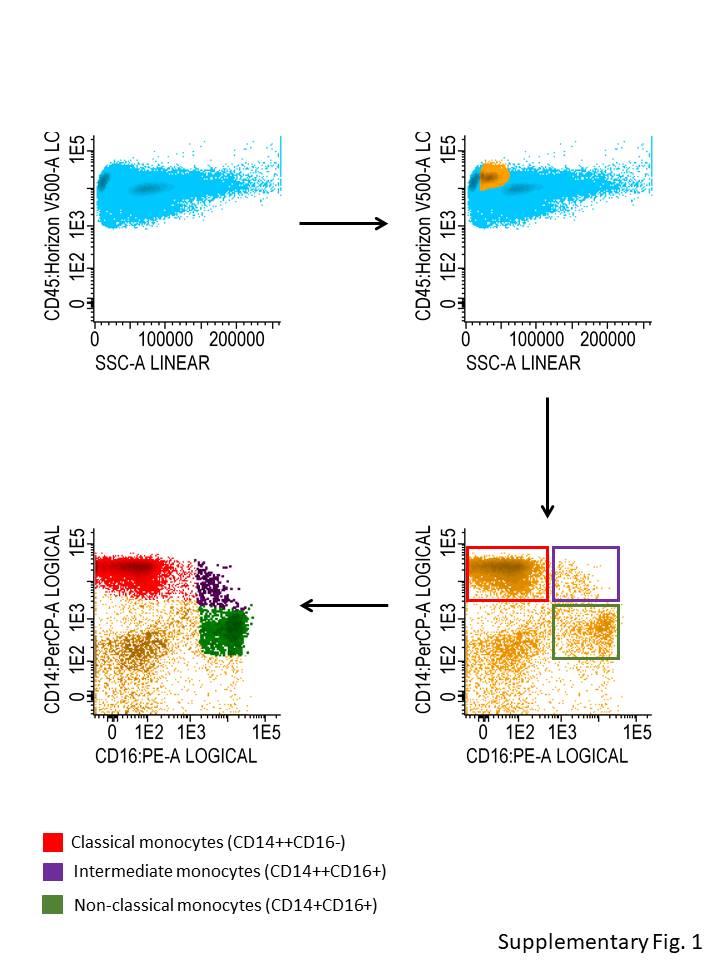

Supplement: Supplementary Figure 1 — Gating strategies for the FACS analysis. Gating strategy used to discriminate between between classical monocytes (CD14++CD16−), intermediate monocytes (CD14++CD16+) and non-classical monocytes (CD14+CD16+). [file Image_1.jpeg]

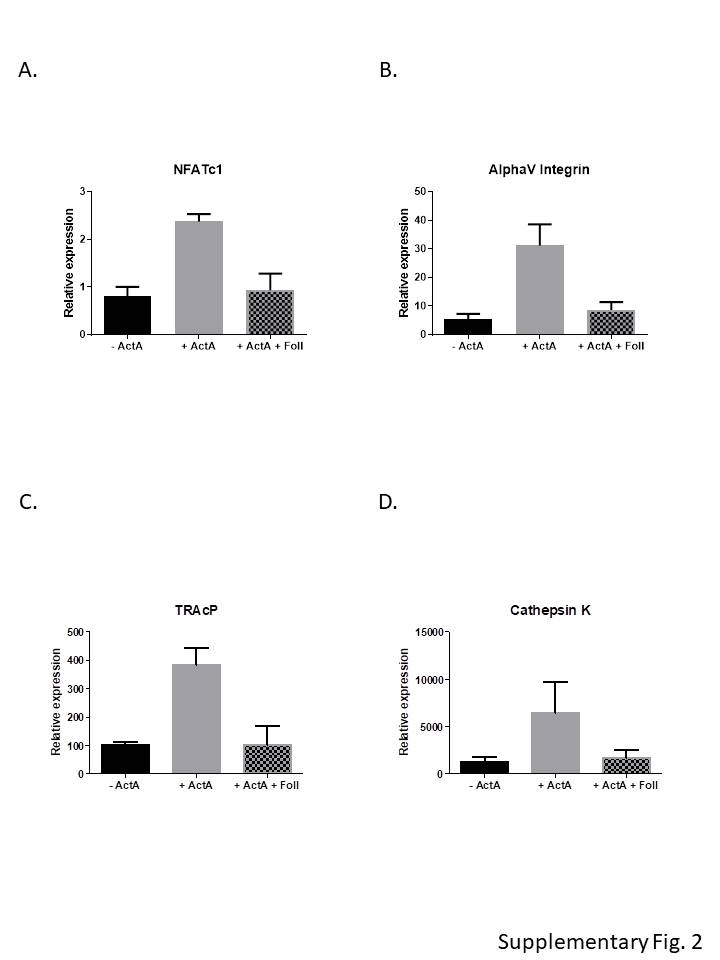

Supplement: Supplementary Figure 2 — Follistatin reduces the Activin-A effect on osteoclast related gene expression. CD14+ cells from pne healthy donor were cultured with M-CSF and RANK-L, without and with Activin-A (50 ng/ml). Experiments were plated in quadruplicate. To block the Activin-A effect a third experimental condition was added where follistatin (500 ng/ml) was present in the cultures. RNA was isolated after 7 days and QPCR was performed. The inductive effect of Activin-A on the gene expression of (A) the early transcription factor NFATc1, (B) the on osteoclasts abundantly expressed AlphaV integrin, (C,D) the osteoclast specific markers TRAcP and Cathepsin K is reduces by follistatin. N = 1, mRNA was isolated from one of confirmative experiments shown in Figure 5, n = 4 wells per condition (Friedmann test with Dunn's multiple comparisons). [file Image_2.jpeg]
