# Supplementary material for: Activin-A Induces Fewer, but Larger Osteoclasts From Monocytes in Both Healthy Controls and Fibrodysplasia Ossificans Progressiva Patients
Source: Front Endocrinol (Lausanne). 2020 Jul 14;11:501. doi: 10.3389/fendo.2020.00501 (PMC7371852; doi:10.3389/fendo.2020.00501)
Supplement: Supplementary Table 1 — Monoclonal antibody clones used in FACS analysis. [file Table_1.docx]

**Supplementary Table 1. Monoclonal Antibody Clones used in FACS analysis.**

| **Monoclonal antibodies** | **Clone** | **Company** |
| --- | --- | --- |
|  |  |  |
| CD45 Krome-Orange | J.33 | Beckman Coulter |
| CD4 APC-H7 | SK3 | BD bioscience |
| CD8 FITC | SK1 | BD bioscience |
| CD19 APC | SJ2C1 | BD bioscience |
| CD14 PercP | MoP9 | BD bioscience |
| CD16 PE | 3G8 | Beckman Coulter |
